# Dermatomyositis and Immune-Mediated Necrotizing Myopathies: A Window on Autoimmunity and Cancer

**DOI:** 10.3389/fimmu.2017.00992

**Published:** 2017-08-21

**Authors:** Audrey Aussy, Olivier Boyer, Nadège Cordel

**Affiliations:** ^1^Normandie University, UNIROUEN, INSERM, U1234, Rouen University Hospital, Department of Immunology, Rouen, France; ^2^Unit of Dermatology and Internal Medicine, Pointe-à-Pitre University Hospital, University of the French West Indies, Fouillole, Pointe-à-Pitre, Guadeloupe

**Keywords:** cancer, myositis, autoimmunity, TIF1gamma, autoantibody

## Abstract

Autoimmune myopathies (myositides) are strongly associated with malignancy. The link between myositis and cancer, originally noticed by Bohan and Peter in their classification in 1975 (1), has been evidenced by large population-based cohort studies and a recent meta-analysis. The numerous reports of cases in which the clinical course of myositis reflects that of cancer and the short delay between myositis and cancer onset support the notion that myositis may be an authentic paraneoplastic disorder. Thus, cancer-associated myositis raises the question of cancer as a cause rather than a consequence of autoimmunity. Among myositides, dermatomyositis and more recently, although to a lesser extent, immune-mediated necrotizing myopathies are the most documented forms associated with cancer. Interestingly, the current diagnostic approach for myositis is based on the identification of specific antibodies where each antibody determines specific clinical features and outcomes. Recent findings have shown that the autoantibodies anti-TIF1γ, anti-NXP2 and anti-HMGCR are associated with cancers in the course of myositis. Herein, we highlight the fact that the targets of these three autoantibodies involve cellular pathways that intervene in tumor promotion and we discuss the role of cancer mutations as autoimmunity triggers in adult myositis.

## Introduction

The link between autoimmunity and cancer has become a topic of unceasing interest over the past decade. Although it is increasingly evident that the risk of cancer is augmented in patients affected by several types of autoimmune diseases (AID), the nature of the interplay between autoimmunity and cancer remains elusive (2–4). One important question here recalls the old egg-and-chicken dilemma: is the autoimmune background in AID a seedbed for cancer development or, alternatively, may cancer cause autoimmunity?

Rheumatic AID such as systemic lupus erythematosus, rheumatoid arthritis, or Sjögren syndrome promote cancer development after several years of chronic inflammation and also exposure to immunosuppressive drugs (2, 5, 6). In the case of lupus for instance, the broadness of cancer type spectrum is striking, including hematological—mostly virus induced—malignancies but also numerous kinds of solid tumors such as vulva, lung, thyroid, and liver cancer (7). Here, the risk of cancer cannot only be ascribed to the sole autoimmune status but also presumably to iatrogenic immunosuppression.

This view is counterbalanced by the examples of paraneoplastic neurological syndromes, systemic sclerosis, and myositis. Paraneoplastic neurological syndromes with anti-neuronal autoantibodies (aAbs) include a most illustrative example, i.e., anti-Hu encephalitis associated with small cell lung carcinoma (SCLC) (8). Anti-Hu aAbs recognize the HuD autoantigen normally restricted to neurons but ectopically expressed on SCLC cells (9). Expression of the immunogenic HuD molecule by SCLC elicits the production of anti-Hu aAb and CD8^+^ cytotoxic T cells, explaining the parallel clinical course of neurological symptoms and SCLC evolution and demonstrating the direct link between cancer and tissue-specific AID (10). Cancer-induced breakage of tolerance can also be caused by tumoral somatic mutations, as recently highlighted by the case of systemic sclerosis associated with anti-polymerase III (POL3) aAbs. The immunogenic peptides generated by *POLR3* gene mutations induce a POL3-specific CD4^+^ T cell response with production of specific antibodies that secondarily target wild-type POL3 by epitope spreading (11).

This present review focuses on the forms of myositides, i.e., dermatomyositis (DM) and immune-mediated necrotizing myopathies (IMNMs) which have been identified as associated with cancer and represent a paradigm of cancer-associated AID.

## DM, Risk of Cancer, and Diagnostic Contribution of Autoantibodies

Autoimmune myopathies or myositides constitute a heterogeneous group of severe acquired myopathies. They are characterized clinically by symmetrical proximal muscle weakness, associated or not with systemic features, and histologically by various levels of myofiber necrosis/regeneration and interstitial mononuclear infiltrates. Clinical and histopathological patterns define different diseases: polymyositis, DM, overlap myositis, sporadic inclusion-body myositis, and IMNM (12–17).

**Figure 1:**
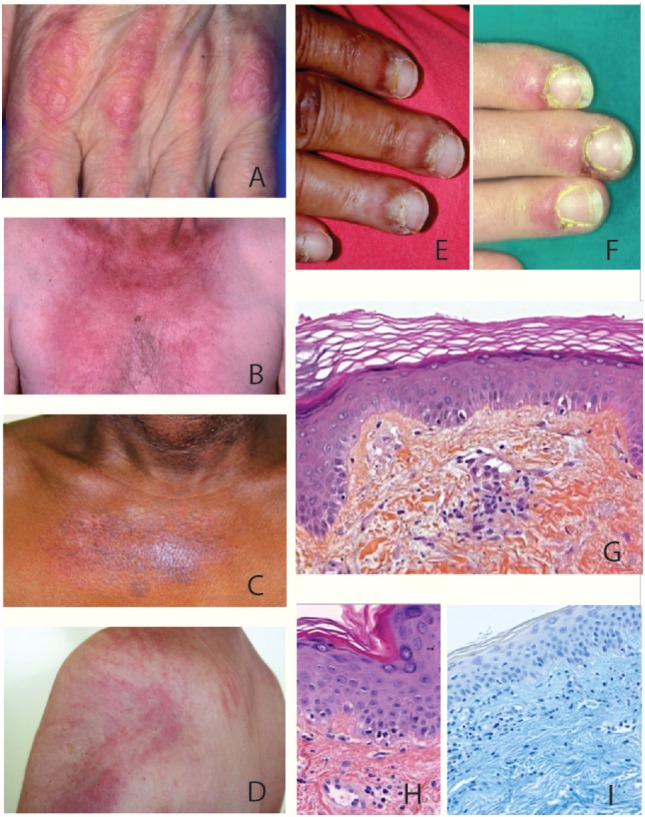
Clinical and histological features of dermatomyositis (DM). **(A)** Gottron’s sign: erythematous scaly papules over the metacarpophalangeal joints. **(B)** «V sign» in a white European male patient with DM. **(C)** Poikiloderma (i.e., erythema, atrophy, variable pigmentary changes) on the upper trunk of an African Caribbean female patient with DM. **(D)** Typical centripetal flagellate erythema affecting the upper trunk of a male patient with DM. **(E,F)** Periungual erythema and telangiectatic capillary loops in patients with DM. **(G–I)** Histological feature of a Gottron’s papule. **(G)** Slight hyperkeratosis, basal cell vacuolar degeneration, upper dermal edema, and perivascular inflammatory cell infiltrate with enlarged capillaries (HES staining, ×20). **(H)** DM interface dermatitis with vacuolar changes of the basal cell layer, perivascular inflammatory cell infiltrate with capillary dilatation, endothelial cell turgescence, and pigmentary incontinence (HES staining, ×40). **(I)** Positive alcian-blue staining attesting dermal mucin deposits (×20).

Dermatomyositis affects both adults and children among all ethnic groups with an unbalanced 1/2-sex-ratio in favor of women. Its annual incidence varies from 1.9 to 7.7 cases per million inhabitants according to data in the literature with a peak of frequency in 40–60-year-old adults and in 5–14-year-old children (13, 18). The appearance of specific cutaneous manifestations is typical of DM and is among its diagnostic criteria. Cutaneous manifestations typically consist of erythematous scaly papules over the metacarpophalangeal knuckles (Gottron’s papules) (Figure 1A); a symmetrical reddish-violet periorbital edema that predominates on the upper eyelids (heliotrope erythema) but may affect the rest of the face; lupus-like erythema which involves low neck (V sign) (Figure 1B), shoulders (shawl sign), extensor surfaces of the limbs, dorsal side of hands and fingers and scalp; poikiloderma of the upper trunk (Figure 1C); and centripetal flagellate erythema affecting the trunk and or proximal extremities (Figure 1D). Cutaneous manifestations of DM also include non-specific lesions such as (i) vascular lesions, i.e., periungual erythema with telangiectatic capillary loops, nail fold dilated capillaries visible to the naked eye, cuticular hypertrophy (Figures 1E,F), vasculitis, cutaneous necrosis, or Raynaud’s phenomenon, which are more prevalent in the course of juvenile dermatomyositis and (ii) several other dermatological features such as pruritus (present in 30% of DM), photosensitivity, mucinosis, and calcifications, which are more frequent in children rather than in adults, i.e., 30–70 versus 10%. Dermatological particularities of DM have been reported in several ethnic groups. In Afro-Caribbeans, edema of the face is usually predominant whereas in Eurasians, the Wong-type DM which mimicks a pytiriasis rubra pilaris seems to be more frequent (19).

Specific lesions of DM are histologically characterized by an interface dermatitis with basal layer vacuolar changes that are associated in various degrees with hyperkeratosis, epidermal atrophy, basement membrane thickening, upper dermal edema, pigmentary incontinence, mucine deposits, and light perivascular CD4^+^ T lymphocyte infiltrate of the superficial dermis (Figures 1G–I) (20).

Dermatological features usually precede muscle weakness by 3–6 months but may appear several years before. However, muscular manifestations may be absent, defining the amyopathic form of DM with an associated cancer rate, which is theoretically the same as classic DM (21, 22).

**Figure 2:**
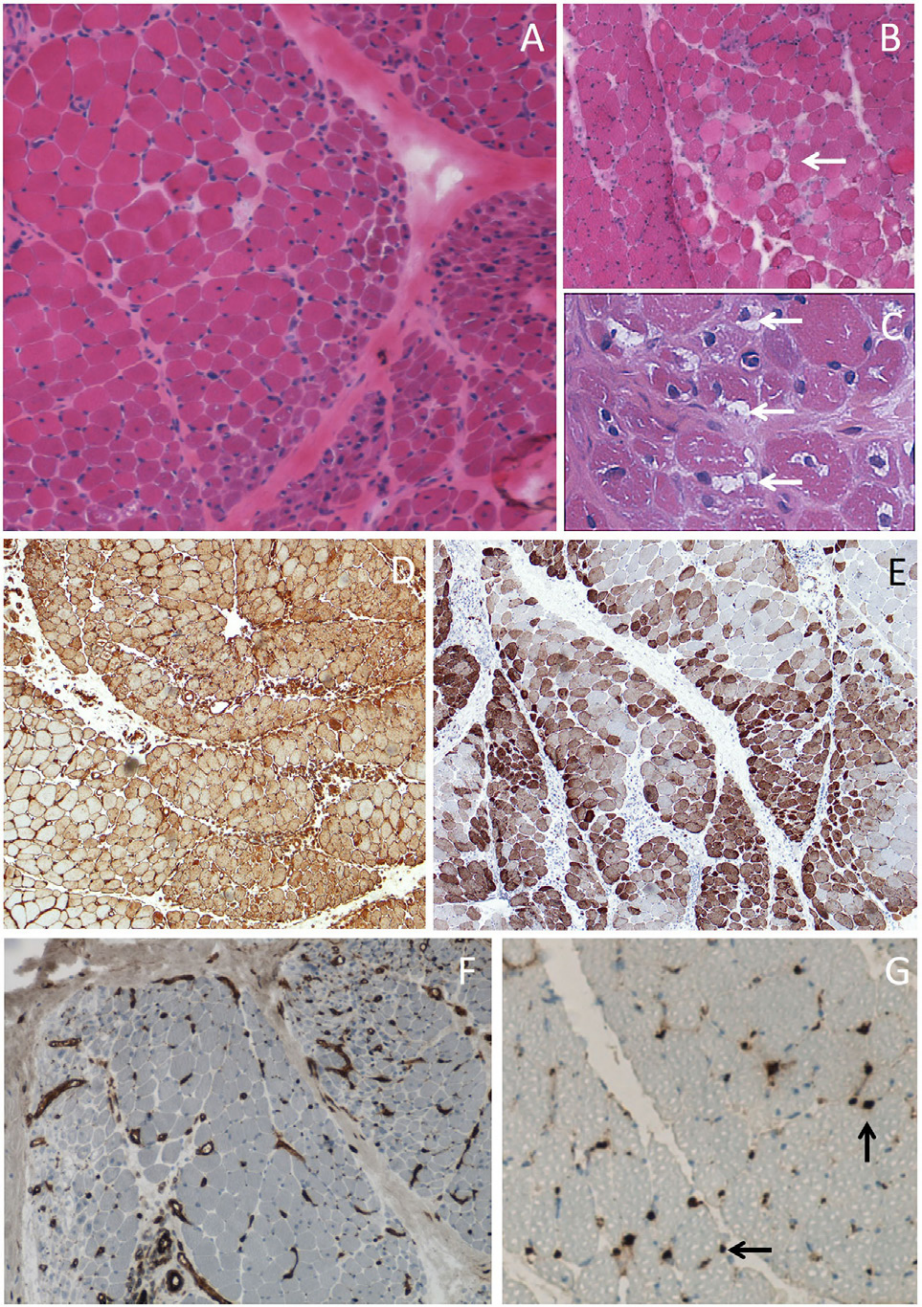
Muscle biopsy of dermatomyositis. **(A)** Perifascicular atrophy. **(B)** Area of contiguous necrotic myofibers (arrow) corresponding to a microinfarct. **(C)** Punch-out vacuoles within myofibers (arrows) assessing focal myosinolysis. **(D)** Ubiquitous myofiber reexpression of MHC-class I with perifascicular reinforcement. **(E)** Neural cell adhesion molecule (NCAM) immunostaining showing large areas of positive myofibers indicating muscle ischemia. **(F)** Platelet endothelial cell adhesion molecule (PECAM) immunostaining for endothelial cells showing marked endomysial capillary drop out. **(G)** Complement activation assessed by the presence of membrane attack complex deposits at the level of endomysial capillaries (arrows). Frozen sections, light microscopy; hematoxylin-eosin **(A–C)**, immunoperoxydase technique **(D–G)**, HLA-ABC **(D)**, CD56/NCAM **(E)**, CD31/PECAM **(F)**, and C5b-9 **(G)**.

Diagnosis of DM is supported by serum creatine kinase (CK) elevation, which mirrors muscle lysis and by electromyographic data, but diagnosis is confirmed by muscle biopsy except for amyopathic DM. Histological examination of muscle tissue (Figure 2) typically shows perifascicular atrophy, necrotic and regenerative muscle fibers, septal and/or perivascular inflammatory cell infiltrate, and endomysial microangiopathy with membranolytic attack complex C5b-9 capillary staining that may also be present in cutaneous lesions. Although these capillary injuries have long been considered as indirect evidence of an initial endothelial target in DM, recent scientific findings demonstrate that capillary lesions might be non-specific in relationship to ischemia-reperfusion injury of perimysial arcade arteries (23). The role for interferons in DM pathogenesis is increasingly evident, since a typical interferon type 1 pathway signature was found in both muscle (24) and skin (25), with a correlation with disease activity (26).

The outcome of DM is variable with benign forms highly responsive to treatment and severe forms associated with cardiac or lung involvement and/or malignancies. In juvenile DM, cancer association is uncommon. Nonetheless, a poor prognosis may be due to the intensity of vasculitis and vascular damage that involves the skin and the digestive system. Calcifications on the areas around joints may also lead to severe functional impairment in children.

Interestingly, several dermatological manifestations of DM such as cutaneous necrosis, skin vasculitis or pruritus, or histological patterns such as leucocytoclastic vasculitis are reported to be associated with cancer whatever the ethnic group (27, 28). Conversely, several features seem to protect against cancer such as Afro-Caribbean ethnicity (29).

Globally, myositides are rare diseases and epidemiologic data remain scarce. Studies are limited by their retrospective character and small population size. Yet, since its first descriptions (30, 31), the association between cancer and myositis has been confirmed by several studies published between 1975 and 2012, reporting a global malignancy rate from 6.7 to 32% (32–35). Importantly, several studies also point to cancer as the main cause of death in cancer-associated myositis patients (34, 36, 37). Yet, it may be difficult to distinguish between cancer on the one hand, and myositis exacerbation and its complications on the other hand, as the actual cause of death. Most observations highlighted the short delay between the onset of myositis and the discovery of cancer. In some cases, cancer even preceded myositis, reinforcing the view that myositis might be the consequence rather than the cause of cancer and even leading to individualizing “cancer-associated myositis” in one classification in 2005 (14). Cancer risk is particularly established in DM. A recent meta-analysis confirmed adult (but not juvenile) DM as a risk-factor of cancer, with a standardized incident ratio (SIR) for occurrence of cancer of 5.5 [4.3–6.7], mostly peaking at 1 year around DM diagnosis (38). A limitation to previous studies, including those reviewed in this meta-analysis, is that the classification of myositis was essentially based on the widely used Bohan and Peter classification which tends to classify IMNM, overlap myositis, and polymyositis in a single category (1). Whereas this classification remains popular for its practical value in DM, progress in the definition of clinical, pathological, and serological patterns has led to newer classifications that are useful for the diagnosis of inclusion-body myositis (12), overlap myositis (14), or IMNM (13). In the light of these different existing classifications for myositis, a new unifying classification would provide a much-awaited tool.

The detection of myositis-specific aAbs (MSA) has proved most helpful in classifying the different forms and predicting the outcome of myositis. Indeed, in clinical practice, MSA define particular clinical ± histological patterns (39). In DM, anti-Mi-2 are classically associated with absence of cancer, sensitivity to treatment and, overall, good prognosis (40–42). Anti-melanoma differentiation antigen 5 (MDA-5) identifies a subgroup of DM patients with interstitial lung disease, necrotizing cutaneous lesions, skin ulcerations, and tender palmar papules while muscle signs are minimal (43–45). Anti-SAE (small ubiquitin-like modifier activating enzyme) has a low prevalence (1–4%) and patients have classic cutaneous signs of DM. Interestingly, a significant but low rate of cancer has been reported in this group (46–48). Anti-TIF1γ (transcriptional intermediary factor 1 gamma) is the leader of cancer-associated aAbs, with a rate of associated malignancy ranging from 60 to 80% of patients (49, 50). Anti-NXP2 (nuclear matrix protein 2) is another biomarker of risk of malignancy in adult patients, with a 30% rate of cancer among DM patients (51, 52).

Besides DM, IMNM is another form of myositis recently suggested to be associated with malignancy (53–55). IMNM may be subdivided in three groups: seronegative, anti-HMGCR (3-hydroxy-3-methylglutaryl-coenzyme-A-reductase), and anti-SRP (signal recognition particle). In contrast to anti-SRP positive patients, one study has recently suggested that, among IMNM, seronegative and anti-HMGCR positive patients have a significantly higher risk of cancer (56).

## Cancer-Associated Myopathies According to Autoantibodies

### DM with Anti-TIF1γ, the Leader in Cancer-Associated Myositis

Anti-TIF1γ was first described in 2006 as an antibody directed against a 155 kDa protein, especially in patients with DM (49, 57). This protein was rapidly identified as TIF1γ. Anti-TIF1γ aAbs scored positive in 20–30% of adult DM and 30–40% of juvenile DM with some differences according to geographical origin (39, 58). HLA DQA1*0301 was associated with anti-TIF1γ DM (49). The high prevalence of cancer was largely confirmed with a rate from 18 up to 80% of adult patients especially in the 2 years surrounding DM (22, 52, 59). A meta-analysis performed by Trallero-Araguás and colleagues estimated a 78% sensitivity and 89% specificity of these antibodies for diagnosing an associated cancer (50). Since 2001, age has been recognized as a risk factor for cancer among DM patients (33). Recently, two publications strengthened the role of advancing age in the increasing risk of cancer among adult anti-TIF1γ positive DM patients (60, 61).

No study found any predominance of one type of cancer; those occurring in adults with DM were generally comparable to those in the general population, stratified by age and sex (breast, lung, colorectal, bladder) as well as to some more rare cancers such as gastric or thymus cancer (59). Anti-TIF1γ DM associates classic but severe cutaneous signs with moderate muscular symptoms, frequent dysphagia but decreased systemic features compared to other DM (60). The specific clinical and histopathological features of anti-TIF1γ DM are summarized in Table 1. Regarding juvenile DM, no increased risk of cancer is observed (62). The median age of DM is 6.8 years of age. Anti-TIF1γ juvenile DM more often presents chronic or polycyclic courses associated with more severe prognosis and profuse cutaneous involvement (63).

**Table 1:** Characteristics of anti-TIF1γ, anti-NXP2, and anti-HMGCR myositis.

	Anti-TIF1γ dermatomyositis (DM)	Anti-NXP2 DM	Anti-HMGCR immune-mediated necrotizing myopathy
Year of discovery of the aAb	2006 (49, 57)	1997 (64)	2010 (65, 66)

Frequency of cancer association in adults	60–80% (22, 52, 59)	24–37.5% (60, 61, 67)	12.9–36% (56, 68, 69)
**Clinical features**			
Skin involvement	Extensive cutaneous signs	Mild skin involvement	Generally not
	Poikiloderma	Frequency of calcinosis	
	Psoriasis-like lesions		
	Scaly erythema of the scalp		
	No calcinosis		
Muscular involvement	Mild weakness	Mild to severe weakness	Mild to severe weakness
			Myalgia
		Frequency of distal weakness	Inconstant dysphagia
	Frequent dysphagia	Myalgia, muscle atrophy	
		Frequent dysphagia mild to severe	
Other characteristics	Peripheral edema	Peripheral edema	High frequency of statin exposure (65, 66, 71)
	Decreased risk of Raynaud phenomenon, arthralgia, and interstitial lung disease (60)	Low frequency of interstitial lung disease (61, 70)	

Histological pattern	Dense C5b-9 deposits on capillaries	Perivascular inflammation	Necrosis
	Presence of vacuolated fibers	Perifascicular atrophy	Muscle fiber regeneration
	Overexpression of MHC class I	Necrosis in patient with peripheral edema (70)	Atrophic fibers
	Perifascicular atrophy		Little or no inflammatory infiltration C5b-9 deposition (13, 72)
	Necrotic/regenerating fibers (59)		
**Target of the aAbs**			
Name	Transcriptional intermediary factor 1 gamma	Nuclear matrix protein 2 or microrchidia 3 (MORC3)	3-hydroxy-3-methylglutaryl-coenzyme-A-reductase
Protein expression	Ubiquitous	Ubiquitous	Ubiquitous
		Immune cells at high level	Liver
Subcellular locations	Nucleus	Nucleus	Endoplasmic reticulum
	Cytosol in part		Peroxisome
Role	TGFβ pathway	Chromatin remodeling	Limiting enzyme for cholesterol synthesis and other mevalonate-dependent pathways (81–83)
	Mitosis	DNA repair	
	Embryonic development	Epigenetic regulation	
	DNA repair	Cell regulation	
	Erythropoiesis	Activation of p53	
	Innate immunity	Calcium homeostasis	
	(73–76)	Bone remodeling (77–80)	

### DM with Anti-NXP2, the Second Actor in Cancer-Associated Myositis

A novel aAb directed against a 140 kDa protein was found in a 1997 cohort of juvenile DM and named anti-MJ (64). The 140 kDa protein was next identified as NXP2, also known as MORC3 (84). Anti-NXP2 aAbs are present in 22–25% of juvenile DM patients and in 1–17% of adults with DM, depending on the method of detection (61, 70). Forms of myositides other than DM may occasionally be associated with anti-NXP2 (67, 84). Cancer was detected in 24–37.5% of adults who scored positive for anti-NXP2 in several retrospective series (60, 61, 67). As for anti-TIF1γ DM, no specific type of tumor was mentioned. Clinical manifestations are partially distinct from anti-TIF1γ DM (Table 1). Indeed, classic cutaneous features are less severe but there is a higher prevalence of calcinosis (70). In addition, muscular involvement is constant and more severe. Similarities between anti-TIF1γ and anti-NXP2 include dysphagia and prevalence of peripheral edema (61, 70). Interstitial lung disease has only been reported in one cohort (85) and Raynaud’s phenomenon is found in 20% of cases (64, 85).

In children, the median age at onset has been calculated at 5.8 years of age (58). Two series found an increased risk of calcinosis in children and a high prevalence of severe muscular involvement with functional disabilities, muscle cramps, and dysphagia but no cancer (Table 1) (39, 51).

## IMNMs, New Players in Cancer-Associated Myositis?

Immune-mediated necrotizing myopathies are a recently described entity, based on specific histological pattern with poor inflammatory infiltrate and presence of significant necrotizing and regenerative fibers (13, 72). As mentioned above, three IMNM subsets have been identified according to serologic status: anti-SRP, anti-HMGCR, or negative serology. Anti-HMGCR and seronegative IMNMs seem to be associated with a higher risk of cancer, with a SIR score of 2.79 and 8.35, respectively (56). Between 13 and 36% of anti-HMGCR positive patients have an associated cancer (56, 68, 69). A genetic study found that HLA-DRB1*11:01 is associated with a higher risk of anti-HMGCR IMNM in both white American and African American adult populations, whereas HLA-DRB1*07:01 seem to be associated with a risk of anti-HMGCR myositis in small series of children (86, 87).

Anti-HMGCR aAbs were discovered in 2010 in a group of patients who developed myositis after exposure to statins, without resolution by stopping statins (65, 66). Histological features corresponded to IMNM (88–90). While the association with statins was confirmed in recent studies, it involved a wide range between 37 and 94% of patients (56, 91). Thus, statins are not necessarily required to develop anti-HMGR myositis. Clinically, patients present severe and acute muscle weakness, dramatically elevated CK level, but extra-muscular disorders are uncommon (65, 66, 71). Anti-HMGCR aAbs have also been identified in the sera of juvenile myositis, without exposure either to statins or cancer. Clinical features in children may wrongly shift toward muscular dystrophy (92).

## Targets of Cancer-Associated Autoimmune Response in Myositis

Intriguingly, all three aAb targets in cancer-associated myositis are involved to some extent in cancer pathogenesis.

### TIF1γ, Encoded by the *TRIM33* Gene

TIF1γ, also known as ecto, RETfused7, or TRIM33, was discovered in 1999 and identified as the third member of the TIF1 protein (after TIF1α and TIF1β) (73). These three proteins belong to the TRIM (tripartite motif) protein family defined by a particular RING-finger domain (93). TIF1 proteins are a subfamily characterized by several domains from 3′ to 5′, including the RING-finger domain, 1 or 2B-boxes, a coiled-coil domain, a plant homeodomain (PHD), and a bromodomain (94). They are involved in multiple critical biological processes. TIF1γ is particularly known for being involved in embryonic development, hematopoiesis, mitosis and cycle regulation, DNA repair, innate and adaptive immunity, osteoblast differentiation, viral transcription, and oncogenesis in case of dysregulation. TIF1γ can exert its role as an E3-ubiquitin ligase, as a histone-binding protein or by sumoylating proteins.

The main interacting pathways of TIF1γ are the TGF-β canonical (Smad4-dependent) and non-canonical (Smad4-independent) pathways (95–97). In the canonical pathway, TIF1γ acts either as a repressor (*via* ubiquitination), a competitor or a partner of Smad4, depending on the cellular context (96). In the canonical TGF-β pathway, the activation of the TGF-β receptor leads to the recruitment of RSmad (Smad 2 or 3) and phosphorylation allows the formation of a RSmad/Smad4 complex, which next enters the nucleus to activate the transcription of targeted genes. During mammalian embryonic development, TIF1γ plays a major role in patterning and polarizing embryonic cells before gastrulation by inhibiting Nodal/Smad4 signaling, which promotes endodermic proliferation (98). Later in embryonic development, Smad4 and TIF1γ cooperate or act redundantly to promote both proliferation and differentiation of neural stem cells and palate development (99, 100). It has been shown that TIF1γ participates in the differentiation of stem cells in collaboration with Smad4, by direct interaction with histone *via* PHD–bromodomain leading to the assembling of Smad4/Smad2–3 complex on targeted genes (101). In adult tissues, TIF1γ promotes the terminal differentiation of mammary gland and lactation by antagonization of Smad4 (102), supports both osteoblast proliferation and differentiation under stimulation of bone morphogenetic proteins (BMP) *via* the activation of a particular RSmad complex (Smad1/5) (103), regulates granulopoiesis in mice (104), and participates in the development of iNKT cells (105). During erythropoiesis, Smad4 and TIF1γ competitively bind to phosphorylated SMad2/3 (RSmad) in response to TGF-β to promote the proliferation and maturation of erythroblasts (106, 107). Otherwise, TIF1γ is involved in the balance between lymphoid and myeloid lineage and protects hematopoietic stem cells from aging (108), through TAL1 and PU1 DNA-binding protein, whose transcriptional activity depend on TGF-β (109, 110). TIF1γ has many other functions in cells, mediated by different pathways and functions. Regarding the innate immune system, TIF1γ directly represses the transcription of the interferon-b gene (*ifnb*) at late phase of macrophage activation (74). Also, it binds multiple chromatin sites in monocyte to promote production of macrophage, binds other chromatin sites in mature macrophage to regulate the responses after toll-like receptor (TLR) activation by lipo-polysaccharide (LPS) (111), and is directly involved in proteasome activation *via* the ubiquitination of DHX33 (112). TIF1γ also has many roles in cell homeostasis; TIF1γ is strongly involved in antiproliferative cellular effect by (i) mediating ubiquitination and then the degradation of LIM-domain-binding protein which is involved in the transcription of cycle activator genes (113) and (ii) interaction with APC/C (anaphase-promoting complex/cyclosome) to promote the alignment and stability of chromosomes during mitosis and to prevent abnormal metaphase–anaphase transition (75, 76). Moreover, TIF1γ is largely involved in DNA repair by recruiting different proteins promoting chromatin relaxation and repair (114, 115). Next, TIF1γ is a strong tumor suppressor by preventing β-catenin degradation (116), epithelial-to-mesenchymal transition (117, 118), and by regulation of the chromatin (119, 120). The role of TIF1γ as a tumor suppressor has been directly shown in chronic myelomonocytic leukemia (121), pancreatic tumor (122–124), hepatocellular carcinoma (125), renal cell carcinoma (126), and non-small cell lung cancer (127), where TIF1γ decreased expression or inactivation promotes proliferation and probably epithelial-to-mesenchymal transition (97–104). Paradoxically, overexpression of TIF1γ is involved in oncogenesis notably in breast cancer, where TIF1γ interferes with TGF-β to promote poorer prognosis (128). Moreover, hyper-expression of TIF1γ has been shown in a significant proportion of colorectal adenocarcinomas (129).

### NXP2, Encoded by the MORC3 Gene

MORC3 (microrchidia3) is a nuclear protein which belongs to a highly conserved nuclear matrix protein family which has recently been identified (77). Four MORC members have been identified and are characterized by three common structural domains (ATPase domain, Zinc finger domain, and coiled-coil domain) involved in chromatin remodeling and epigenetic regulation (77). MORC3 includes two other domains, a nuclear matrix binding site and an RNA binding site, essential for the regulation of transcription (78). Its mRNA expression is relatively ubiquitous but the expression of MORC3 protein is particularly elevated in immune cells (78, 79). MORC3 plays several critical roles in cell regulation, illustrated by an early death at birth or day 1 in MORC3 knockout mice (130). MORC3 promotes the activation of p53, a tumor suppressor inducing cellular senescence in response to oncogenic factors and supports the architecture of the nucleus (78, 130). The ATPase domain of MORC3 interacts with the coiled-coil domain to form homodimers that can bind DNA (131). Zinc finger is implicated in the nucleus localization and binds MORC3 to histone (132). These two mechanisms seem to be involved in DNA repair and epigenetic regulation (77, 80). MORC3 interacts with ROR1, a tyrosine kinase involved in pre-B cell receptor signaling pathway promoting cell proliferation (133). Finally, MORC3 plays a role in the transduction of calcium homeostasis regulator and is involved in bone remodeling (134).

### HMGCR, Encoded by the *HMGCR* Gene

HMGCR is a limiting enzyme from the cholesterol biosynthetic chain, catalyzing the reduction reaction of HMGCoA to mevalonate (81). This enzyme has a large transmembrane domain in the endoplasmic reticular membrane and the harbored cytosolic N and C terminal domain. The catalytic domain is in the C-terminal domain (82, 83). This catalytic domain is the target of both statins and specific autoantibodies. Interestingly, it has been recently shown that its functions were indirectly crucial in many pathways. Indeed, HMGCR positively regulates the growth and migration of glioblastoma cells and could play a role in the metastatic capacities of tumoral cells, as well as other enzymes involved in lipidic metabolism (135). The role of HMGCR and the mevalonate pathway in oncogenesis has been suspected for two decades and cases of tumors have been reported to exhibit higher level and activity of HMGCR (136, 137). In 2010, Clendening et al. (138) confirmed this hypothesis by showing that the dysregulation of HMGCR promotes transformation of normal breast epithelial cells and growth of transformed cells (138). Moreover, different levels of HMGCR are associated with different response to radiotherapy in bladder cancer (139) and upregulation of HMGCR is also known to promote proliferation and migration of malignant cells in both glioblastoma and gastric cancer (135, 140). *In vitro* inhibition of HMGCR performed by specific miRNA limits proliferation, invasion, and metastasis process of breast cancer cells (141).

## TIF1γ, NXP2, and HMGCR: Targets of an Antitumoral Response?

In the model proposed by Joseph and colleagues in systemic sclerosis, the immune response directed against POL3 was initially an antitumor immune response. Oncogenesis results from random and additive mutations on several genes some of which are involved in mitotic checkpoint, DNA repair, or differentiation, under the effect of viruses, oncogenes, or radiation. Later, tumoral cells can over-express, ectopically express, or express mutated forms of distinct proteins. The immune system may recognize these newly synthetized forms as neoantigens, which may ultimately lead to T cell and B cell responses (142, 143).

**Figure 3:**
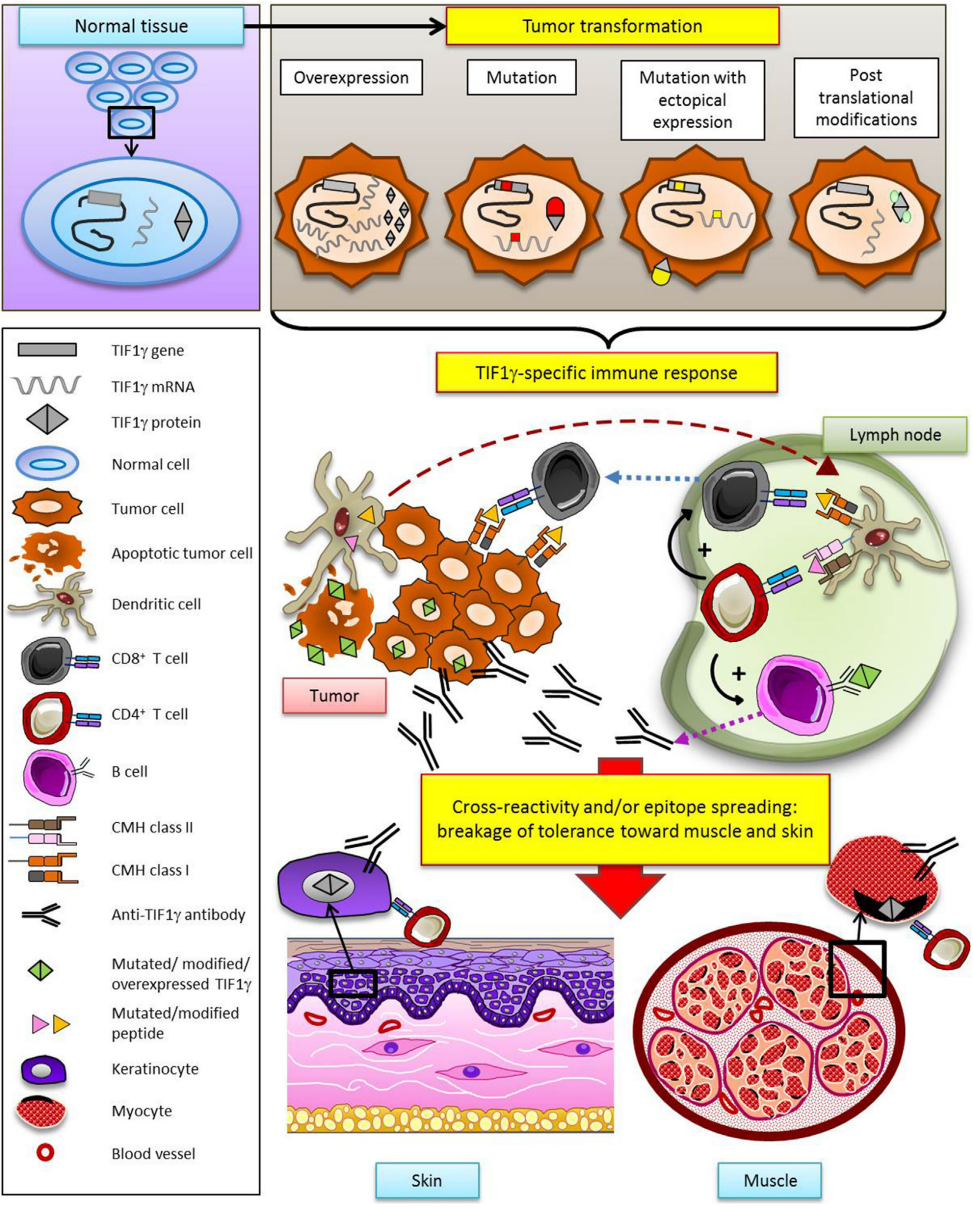
Hypothetical model of specific antitumoral response as a trigger of dermatomyositis through the example of TIF1γ. According to this model, TIF1γ (or NXP2, or HMGCR) is modified in the tumor (gene mutation, overexpression, ectopic expression, posttranslational modification), leading to the development of a TIF1γ- (or NXP2-, or HMGCR-) specific T and B cell antitumor response. Secondarily, breakage of tolerance results from cross-reactivity and/or epitope spreading, promoting a response against muscle and skin.

In many human cancers, TIF1γ is considered as a tumor suppressor by inhibiting the TGF-β pathway. HMGCR also seems to be involved to some extent in oncogenesis through its role in metabolic pathways. Today, NXP2 is not directly known to be involved in cancer but it interacts with the well documented p53 tumor suppressor. Therefore, the targets of these three MSA appear to be proteins involved in cellular pathways that intervene in tumor promotion. Thus, it is reasonable to hypothesize that somatic mutations of TIF1γ, NXP2, or HMGCR genes in tumors may provoke a specific antitumoral immune response which may secondarily extend to the target organs of myositis (muscle, skin) by cross-reactivity and/or a process of epitope spreading (Figure 3). It is tempting to speculate that absence of cancer in some myositis patients may result from an efficacious antitumoral immune response: in this view, myositis might be the immunological price to pay for tumor eradication.

The hypothesis developed herein implies that the abovementioned antitumoral response provokes/sustains myositis. How this response could promote myositis is unclear. Indeed, the three TIF1γ, NXP-2, and HMGCR proteins are ubiquitously expressed, raising the question of how they could be muscle/skin-specific. In this regard, it should be remembered that most if not all MSAs, associated with cancer or not, are specific for ubiquitously expressed, intracellular proteins such as t-RNA synthetases, MDA-5, and signal recognition particles.

Some elements support the direct pathogenicity of aAbs in some forms of myositis. For instance, aAb level correlates with clinical evolution in IMNM (72, 144–146). The pathogenic effect of aAbs is particularly real for anti-HMGCR aAb. Indeed, Arouche-Delaperche and colleagues recently demonstrated that anti-HMGCR aAbs promoted muscle atrophy and impaired regeneration and expression of inflammatory cytokines (147). A similar effect could be expected for anti-TIF1γ and NXP2 aAbs. TIF1γ has been shown to be over-expressed in regenerating muscle (148), which could explain the recognition of TIF1γ in muscle by aAb.

## The Therapeutic Dilemma of Cancer-Associated Myositis

Most myositis clinical trials exclude patients with cancer, limiting the spectrum of our therapeutic knowledge. While beneficial for treating myositis, immunosuppression is of difficult use in the presence of cancer. Reciprocally, myositis alters the patient’s condition and complicates the therapeutic strategy, i.e., administering antitumoral drugs or performing surgery. Guidelines will be required to help manage patients in this context. The hypothesis of myositis triggered by cancer, rather than the opposite, supports a therapeutic strategy of performing an active antitumoral treatment compatible with the patient’s myositis status. Among new cancer therapies, immune checkpoint inhibitors may expose the patient to an exacerbation of autoimmunity that may yield a myositis flare. Since aAbs may be directly involved in myositis pathogenesis, therapeutic trials evaluating plasma exchanges are welcome.

## Conclusion

Taken together, available data point to a unicist view of cancer-associated myositis, in which AID may result from an antitumoral response. This response may be triggered by mutations, overexpression, or posttranslational modification of the autoantigen in tumor. Elucidating these mechanisms will provide strong clues to better understand the potential role of cancer as a cause of autoimmunity.

## Author Contributions

The first version of this review was written by AA and NC. OB revised it critically for important intellectual content. All authors approved the version to be published.

## Conflict of Interest Statement

The authors declare that the research was conducted in the absence of any commercial or financial relationships that could be construed as a potential conflict of interest.
